# Predicting poststroke dyskinesia with resting-state functional connectivity in the motor network

**DOI:** 10.1117/1.NPh.10.2.025001

**Published:** 2023-04-04

**Authors:** Shuoshu Lin, Dan Wang, Haojun Sang, Hongjun Xiao, Kecheng Yan, Dongyang Wang, Yizheng Zhang, Li Yi, Guangjian Shao, Zhiyong Shao, Aoran Yang, Lei Zhang, Jinyan Sun

**Affiliations:** a Foshan University, School of Mechatronic Engineering and Automation, Foshan, China; b Beijing Rehabilitation Hospital of Capital Medical University, Department of Traditional Chinese Medicine, Beijing, China; c Chinese Institute for Brain Research, Beijing, China; d Capital Medical University, School of Biomedical Engineering, Beijing, China; e Foshan University, School of Medicine, Foshan, China

**Keywords:** near-infrared spectroscopy, stroke, motor network, machine learning, dyskinesia

## Abstract

**Significance:**

Motor function evaluation is essential for poststroke dyskinesia rehabilitation. Neuroimaging techniques combined with machine learning help decode a patient’s functional status. However, more research is needed to investigate how individual brain function information predicts the dyskinesia degree of stroke patients.

**Aim:**

We investigated stroke patients’ motor network reorganization and proposed a machine learning-based method to predict the patients’ motor dysfunction.

**Approach:**

Near-infrared spectroscopy (NIRS) was used to measure hemodynamic signals of the motor cortex in the resting state (RS) from 11 healthy subjects and 31 stroke patients, 15 with mild dyskinesia (Mild), and 16 with moderate-to-severe dyskinesia (MtS). The graph theory was used to analyze the motor network characteristics.

**Results:**

The small-world properties of the motor network were significantly different between groups: (1) clustering coefficient, local efficiency, and transitivity: MtS > Mild > Healthy and (2) global efficiency: MtS < Mild < Healthy. These four properties linearly correlated with patients’ Fugl-Meyer Assessment scores. Using the small-world properties as features, we constructed support vector machine (SVM) models that classified the three groups of subjects with an accuracy of 85.7%.

**Conclusions:**

Our results show that NIRS, RS functional connectivity, and SVM together constitute an effective method for assessing the poststroke dyskinesia degree at the individual level.

## 1 Introduction

Stroke has been ranked as the second leading cause of death and disability-adjusted life-years worldwide. It has a high incidence, disability rate, recurrence rate, mortality rate, and financial burden.^1^ Motor, language, and swallowing dysfunctions are commonly observed in stroke survivors. The most common function deficit after a stroke is motor dysfunction, which can leave patients disabled and have a negative impact on their quality of life. Rehabilitation can assist in restoring some of the lost limb functions.^2^^,^^3^ At present, various clinical rating scales are usually used to evaluate the motor function of stroke patients, such as the Fugl-Meyer Assessment (FMA), Berg Balance Scale, and Brunnstrom. Although these methods are well validated and acknowledged, they are still limited by physician subjectivity, large time consumption, and low resolution.^4^

To provide accurate and personalized rehabilitation programs, it is essential to assess limb functions automatically at the individual level.^5^ With the advancement of sensor technology and machine learning algorithms, objective and accurate data can now be gained to provide physicians with additional information about patients’ motor functions.^6^^–^^8^ For example, Zhang et al.^9^ collected upper limb position data from stroke patients and healthy controls with a posture sensor and differentiated among individuals with different types of upper limb dysfunctions with machine learning algorithms. In another study, Xi et al.^10^ demonstrated the effectiveness of using surface electromyography (EMG) in rehabilitation monitoring by combining time, time-frequency, and entropy domain features and the support vector machine (SVM) technique. The present quantitative assessment of motor function in stroke patients focuses primarily on movements, gaits, and EMG signals. However, the origins of motor dysfunction in stroke patients are damages to the brain’s neural circuit. It is quite necessary to evaluate patients’ motor function based on brain functional activity.

Functional magnetic resonance imaging (fMRI), near-infrared spectroscopy (NIRS), and electroencephalography are effective and non-invasive methods for studying brain functions and recovery in stroke patients.^11^^–^^13^ After a stroke, state-specific and general brain function reorganizations coexist and interact.^14^^–^^17^ Resting-state (RS) connectivity can provide useful information about post-stroke brain functions and recovery.^18^^–^^20^ Using fMRI and the graph theory, Cheng et al.^21^ studied the reorganization of whole brain RS functional connectivity (RSFC) in stroke patients and found a positive correlation between the characteristic path length and patients’ motor function. Wang et al.^22^ also used the graph theory and found that RSFC reorganization in the motor system was associated with stroke patients’ dyskinesias. Both RSFC and task-related effective connectivity can explain patients’ motor dysfunction.^23^ These indicate that RS has great potential for assessing post-stroke motor function.

Neuroimaging techniques combined with machine learning approaches are helpful with decoding patients’ functional status at the individual level and realizing precision medicine.^24^^–^^26^ For example, Jane et al.^27^ applied Gaussian process regression to decode upper limb motor impairment with a structural MRI in chronic stroke patients, and the correlation between the actual and model predicted scores was 0.66 (RMSE = 0.79). Rehme et al.^28^ classified patients with hand motor deficits compared with controls and nonimpaired patients with an 82.6% to 87.6% accuracy by combining the whole-brain RS connectivity and SVM. However, more research is required to investigate how individual brain function information can be used to predict varying degrees of motor dysfunction in stroke patients. In this study, we aim to develop a method for classifying the dyskinesia degree in stroke patients by combining NIRS, RSFC, and machine learning.

NIRS was used to collect the RS brain activity in the motor cortex. NIRS has become an important tool in stroke research due to its non-invasive nature, low cost, portability, little constraint for subjects, great anti-interference capability, and minimal environmental requirement.^29^^–^^31^ The motor network’s topology was analyzed with graph theory. We investigated the motor network reorganization of stroke patients and how this reorganization was linked to their motor function. Then, the stroke patients were divided into two groups based on FMA scores: the mild dyskinesia group (referred to as Mild) and the moderate-to-severe dyskinesia group [referred to as moderate-to-severe (MtS)]. We tried to establish models that used the selected brain network properties as features to categorize various groups of subjects.

## 2 Methods

### 2.1 Subjects

Thirty-one stroke patients enrolled from the Chinese Medicine Rehabilitation Center of Beijing Rehabilitation Hospital Affiliated to Capital Medical University participated in this study. The inclusion criteria were as follows: (1) MRI or CT confirmation of the first unilateral stroke, (2) age: 30 to 80 years, (3) modified Ashworth score ≤2 points, (4) Glasgow coma ccale score≥8, and (5) ability to maintain the sitting position for at least 6 min. The exclusion criteria were as follows: (1) previous experience with mental illness or taking antipsychotic medication or (2) having any of the following conditions: congestive heart failure, respiratory failure, deep vein thrombosis of the lower extremities, malignant progressive hypertension, active liver disease, or severe liver or kidney insufficiency. FMA was used to evaluate the patient’s motor function. According to the FMA scores, stroke patients were divided into two groups: Mild (15 patients, FMA≥85) and MtS (16 patients, FMA<85). We also recruited 11 healthy people, ranging in age from 48 to 59 years (mean ± SD: 50.91±3.09), as the control group. All subjects signed an informed consent form and were informed of the basic requirements and procedures prior to the experiment. The Ethics Committee of Beijing Rehabilitation Hospital Affiliated to Capital Medical University reviewed and approved this study. The registration number for the clinical trial is ChiCTR2000040137.

### 2.2 Data Collection

**Fig. 1:**
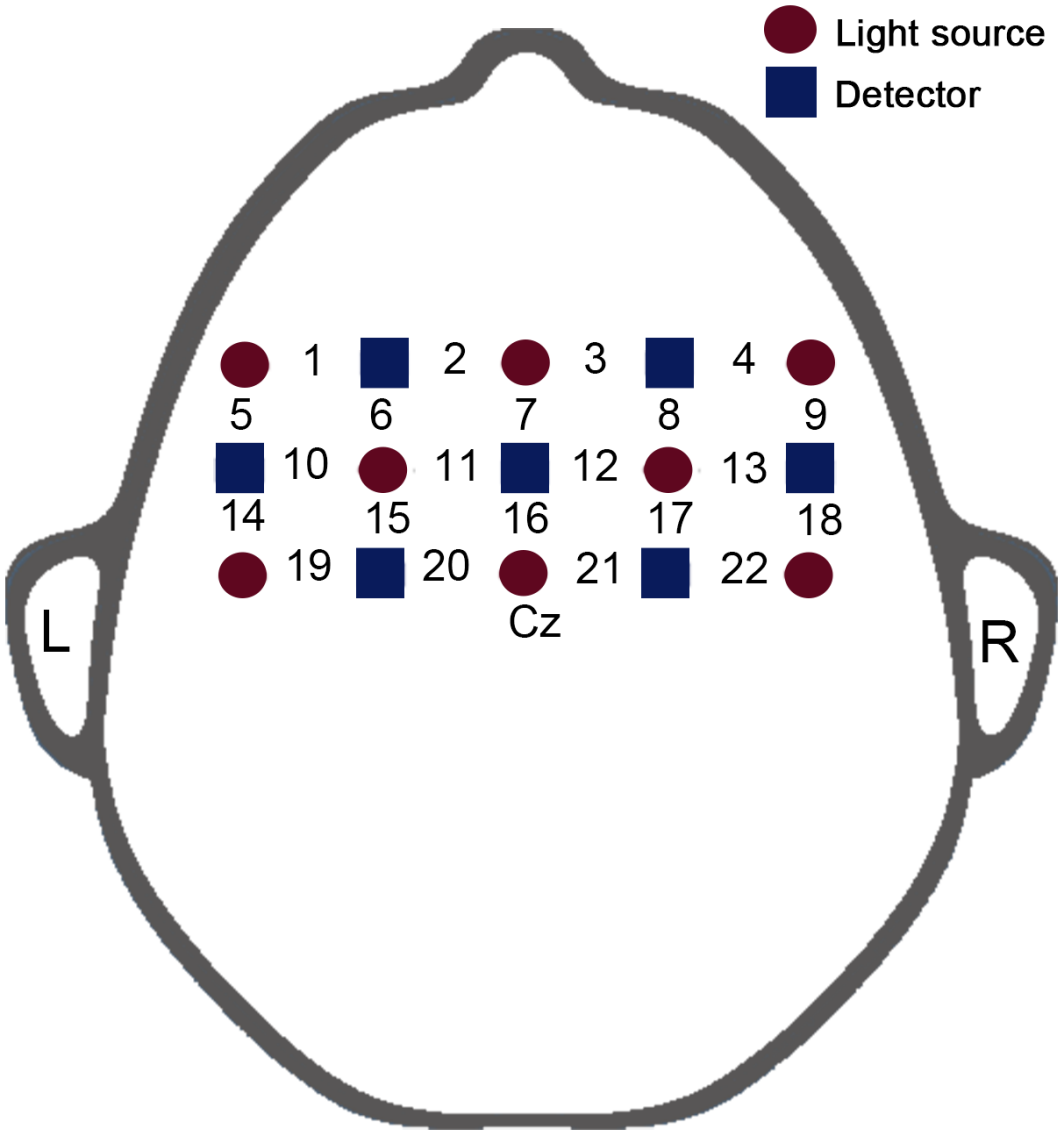
NIRS-measured brain area schematic diagram.

We used the ETG-4000 (Hitachi, Japan), a 22-channel NIRS imaging device, to measure the hemodynamic signals in the motor cortex from subjects in the RS. The NIRS cap consisted of 7 detectors and 8 emitters (light sources), making up 22 channels. The distance between the detector and emitter was 30 mm, and the brain region below each channel (i.e., midpoint between detector and emitter) was the main detection area for the channel. Referring to the 10/20 electrode placement system, Cz was used as the anchor point for placing the NIRS cap, and the lower edge of the NIRS cap was parallel to the coronal axis (Fig. 1). According to previous studies, this source-detector placement covers the motor-related cortex area, including the premotor cortex, the supplementary motor cortex, and the primary motor cortex.^32^^–^^35^ The experiment was conducted in a dark, quiet setting. To reduce movement artifacts, participants were instructed to close their eyes, remain conscious, and refrain from moving their bodies throughout the experiment. With a sampling rate of 10 Hz, NIRS data were obtained for 6 min.^36^

### 2.3 Data Processing

By measuring the attenuation variations of near infrared light at two wavelengths (695 and 830 nm), ETG-4000 used the modified Beer-Lambert law to calculate two cerebral hemodynamic signals: the concentration changes of oxygenated hemoglobin ($\Delta [{\mathrm{HbO}}_{2}]$) and deoxygenated hemoglobin (Δ[Hb]). The hemodynamic signals were pre-processed in Matlab. The pre-processing involved the following steps: (1) detecting and correcting motion artifacts with the standard deviation and spline interpolation method, (2) removing frequency noise and low-frequency baseline drift with a second order band-pass Butterworth filter (0.01 to 0.1 Hz),^37^ (3) reducing the superficial interference with the common average reference spatial filtering,^38^^,^^39^ and (4) excluding the first and last 5 s of data to ensure a stable signal. Most subjects had 350-s of data for subsequent brain network analyses, whereas nine participants used 180-s of data for brain network analyses due to residual artifacts. Compared with Δ[Hb], $\Delta [{\mathrm{HbO}}_{2}]$ has a higher signal-to-noise ratio and a higher correlation with the blood oxygen concentration dependence signal.^40^ Thus, only the ${\mathrm{HbO}}_{2}$ data were analyzed in this paper, and the corresponding results of Hb are shown in the Supplementary Material.

### 2.4 Correlation Matrix and Graph Construction

We used the 22 NIRS channels as network nodes and calculated Pearson correlation coefficients between each pair of channels to obtain a correlation coefficient matrix of 22×22. Fisher’s Z transformation was used to convert the correlation matrix into a Z-value matrix to improve the normality.^41^ The matrix’s diagonal value was set to zero. Finally, the Z-value matrix was converted into the corresponding binary matrix by sparseness with different thresholds. All analyses were carried out over a range of thresholds because there is no “correct” threshold.^42^ We selected the threshold ranges of 0.3 to 0.7 with a step size of 0.01 to rarefy the network. 0.3 was chosen to exclude the low-level correlation in topology, and 0.7 was chosen to reduce the data splitting.^43^ The absolute values of Z-scores in the matrix were sorted from smallest to largest, and those with absolute values greater than the threshold were set to 1 in the corresponding binary matrix, and the others were set to 0. For example, when the threshold was set as 0.4, the first 60% absolute values of Z were set to 1 in the corresponding binary matrix, and the others were set to 0. A value of 1 indicated that two channels were connected, that is, an edge existed between these two channels. Constructing graphs in this manner guaranteed the comparison of different groups’ topological properties under the same connection number.

### 2.5 Small-World Properties

To analyze the motor network characteristic, we used the brain connectivity toolbox to calculate the following small-world properties of each binary network:^44^ clustering coefficient (C), characteristic path length (L), transitivity (T), local efficiency (LE), global efficiency (GE), normalized clustering coefficient (γ), normalized characteristic path length (λ), and small-worldness (δ).

A node’s clustering coefficient ($C_{i}$) represents the degree of connectivity between the node and its neighbors. The network’s clustering coefficient (C) is the average of all node clustering coefficients [Eq. (1)], and it reflects the entire network’s degree of local clustering and segregation; it is given as

$$C=\frac{1}{m}\overset{m}{\underset{i=1}{\sum }}C_{i}=\frac{1}{m}\overset{m}{\underset{i=1}{\sum }}\frac{2e_{i}}{K_{i}(K_{i}-1)},$$ (1)

where $C_{i}$ represents the clustering coefficient of node i, $e_{i}$ represents the number of neighboring nodes directly connected to node i, $K_{i}$ represents the node degree, and m is the total number of nodes.

The characteristic path length (L) is the average of all shortest paths between pairs of nodes [Eq. (2)] and reflects the overall routing efficiency and integration of the network. A shorter L indicates faster information propagation. L is calculated as

$$L=\frac{1}{m(m-1)}\underset{i\neq j\in m}{\sum }d_{i,j},$$ (2)

where the shortest path length $d_{i,j}$ refers to the minimum number of edges required to connect nodes i and j.

The global efficiency (GE) reflects the network’s global transmission capacity, which is the average of efficiencies between nodes and is calculated as

$$\mathrm{GE}=\frac{1}{m(m-1)}\underset{i\neq j\in m}{\sum }\frac{1}{d_{i,j}}.$$ (3)

The local efficiency (LE) is the mean efficiency of the subgraph $G_{i}$, which consists of all of the neighboring nodes for each node and is calculated as

$$LE=\frac{1}{m(m-1)}\overset{m}{\underset{i=1}{\sum }}E(G_{i}).$$ (4)

Transitivity (T) is the ratio of triangles (closed triples) to triples (three nodes that have connections) in the network. It is given as

$$T=\frac{\text{Triangle number}}{\text{Triple number}}.$$ (5)

To check whether the motor network had a small-world characteristic, its clustering coefficient and characteristic path length were compared with those from a random network with a similar node number and degree distribution. In general, we expected the small-world network to have ratios of γ>1, λ≈1, and δ>1

$$\gamma =C/C^{\text{rand}},$$ (6)

$$\lambda =L/L^{\text{rand}},$$ (7)

δ=γ/λ, (8)

where $C^{\text{rand}}$ and $L^{\text{rand}}$ are the averaged C and L of 100 random networks, respectively.

To overcome the threshold influence on statistical analyses and modeling, we calculated the area between the topological property curve and the X-axis by the numerical integration method to get the area under the curve (AUC).^45^ The AUC can provide an aggregated scalar that is not affected by the choice of a single threshold^46^ and is sensitive to the network topology change.^47^^,^^48^ We compared the AUC indicators between groups, analyzed the correlation between patients’ FMA and AUCs of topology properties, and used the AUC indicators as features in SVM.

### 2.6 Relation Between Network Properties and Patients’ Motor Function

To explore the relationship between patients’ motor function and the motor network properties, we calculated the Partial correlations between patients’ FMA and the AUCs of topology properties with age and gender as control variables.

### 2.7 SVM Classification

We used SVM to classify Mild patients, MtS patients, and Healthy controls in Python. SVM is a supervised machine learning algorithm. Through the selection of function subsets and corresponding discriminant functions, the actual risk of the SVM classifier is minimized according to the structural risk minimization criterion. SVM’s optimal decision plane is dependent only on the position and number of support vectors, which can perform well in the case of small samples. Essentially, SVM is a two-group classifier. It is possible to use one-versus-rest (OVR) or one-versus-one strategies for multigroup classification.

We used the AUCs of eight topological properties as alternative characteristics and used the recursive feature elimination method to select the features. The performance of SVM was validated using a nested cross-validation (CV) process with an outer and an inner loop. In the outer loop, we used leave-one-out CV, with one subject as the test sample and the remaining subjects as the training samples that were input to the inner loop. In the inner loop, we used 10-fold CV and used grid search to optimize the optimal model parameters. Specifically, all possible parameter combinations were enumerated by the grid search, and each parameter combination was trained 10 times. Then we selected the parameter combination with the best performance in the inner loop and applied it to the model evaluation in the outer loop. The OVR approach was used for the multigroup classification, and we calculated the sensitivity and specificity of each classifier.

### 2.8 Statistical Analyses

The analysis of variance (ANOVA) with age and gender as covariables and group (Healthy, Mild, and MtS) as the between-subject factor was used to examine the differences in network topological properties among groups. Then posthoc T-tests (LSD correction) were performed for multiple comparisons. The Shapiro-Wilk test was used to determine whether the data had a normal distribution, and the Levene test was used to determine whether the variance was homogeneous. Statistical Package for the Social Sciences (SPSS) was used for statistical analyses, and the level of statistical significance was set to 0.05.

## 3 Results

### 3.1 Clinical Results

In this study, stroke patients were at 1.2±1.04 months after a stroke. As shown in Table 1, there was no significant difference in gender among the three groups, but there was a significant difference in age between groups. In the later analyses, we took both age and gender as control variables to eliminate their influence on the results. Lesion side and time after stroke onset were not significantly different between the Mild and MtS groups, and the FMA score was evidently lower for the MtS group than the Mild group (Table 1).

**Table 1:** Subject characteristics.

	Stroke patients		Healthy subjects	p (χ12-test or ^2^ANOVA)
	Mild	MtS		
Age (years)	65.26 ± 8.12	59.68 ± 10.98	50.91 ± 3.09	^2^<0.01
Gender (male/female)	13/2	12/4	10/1	^1^0.212
Lesion side (left/right)	7/8	7/9		^1^0.870
FMA	94 ± 5.05	40.38 ± 26.22		^2^<0.001
Time after stroke onset (month)	1.08 ± 0.83	1.33 ± 1.21		^2^0.512

### 3.2 Small-World Properties

**Fig. 2:**
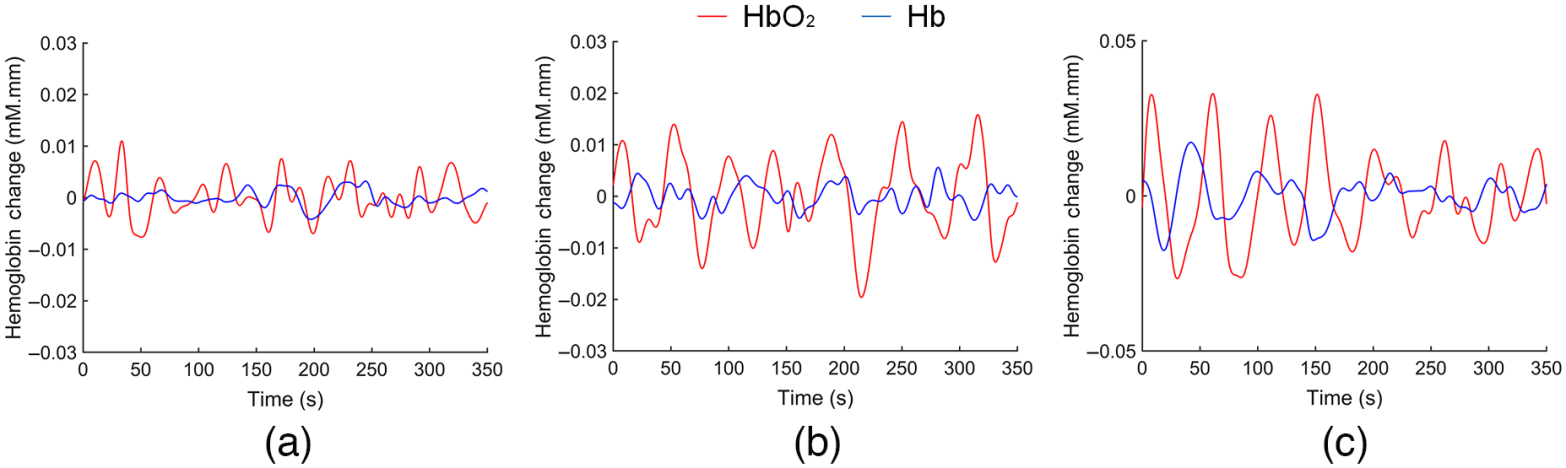
Time course of $\Delta [{\mathrm{HbO}}_{2}]$ and Δ[Hb] at a typical channel (Ch13) from one Healthy subject (a), one Mild patient (b), and one MtS patient (c).

**Fig. 3:**
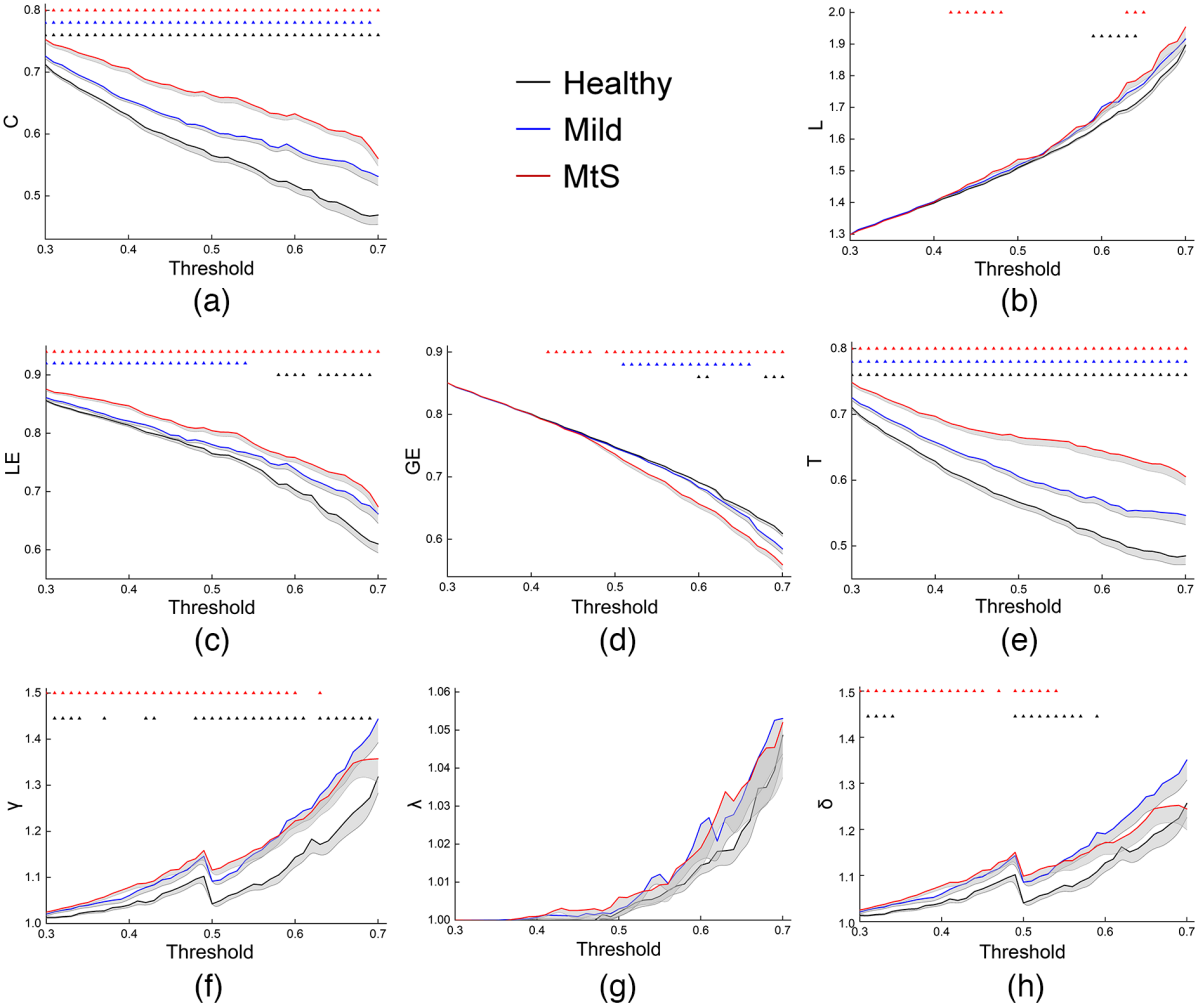
Mean C (a), L (b), LE (c), GE (d), T (e), γ (f), λ (g), and δ (h) for the three groups under each threshold. The shaded part indicates the standard error (SEM). The black, blue, and red small triangles represent significant differences between Healthy and Mild groups, Mild and MtS groups, and Healthy and MtS groups. (p < 0.05, FDR correction) under this threshold, respectively.

The hemodynamic signals at a typical channel from one Healthy subject, one Mild patient, and one MtS patient are shown in Fig. 2. $\Delta [{\mathrm{HbO}}_{2}]$ was used for calculating the motor network’s small-world properties. The trends of the motor network’s small-world properties for the three subject groups are depicted in Fig. 3. The majority of node pairs had connections when the threshold was low. When the threshold increased, some connections were lost, which led to a C decrease. Therefore, connections between node pairs had to go through more nodes, resulting in the L increase. The number of closed triples, as well as the local and global information transmission efficiency, decreased. High clustering (γ>1), small characteristic path (λ≈1), and small-world characteristics (δ>1) were observed in all three groups.

Our first goal was to study the patients’ motor network reorganization. Thus, we examined how the three groups’ network properties differed from each other. As shown in Fig. 3, C, LE, and T for the Mild group were lower than those for the MtS group and larger than those for the Healthy group. This was observed across a wide range of thresholds. At certain thresholds, GE for the Mild group was larger than that for the MtS group and lower than that for the healthy group. L, γ, and δ were greater in both the mild and MtS groups than in the healthy group under some thresholds. There was almost no intergroup difference in λ among the three groups.

**Fig. 4:**
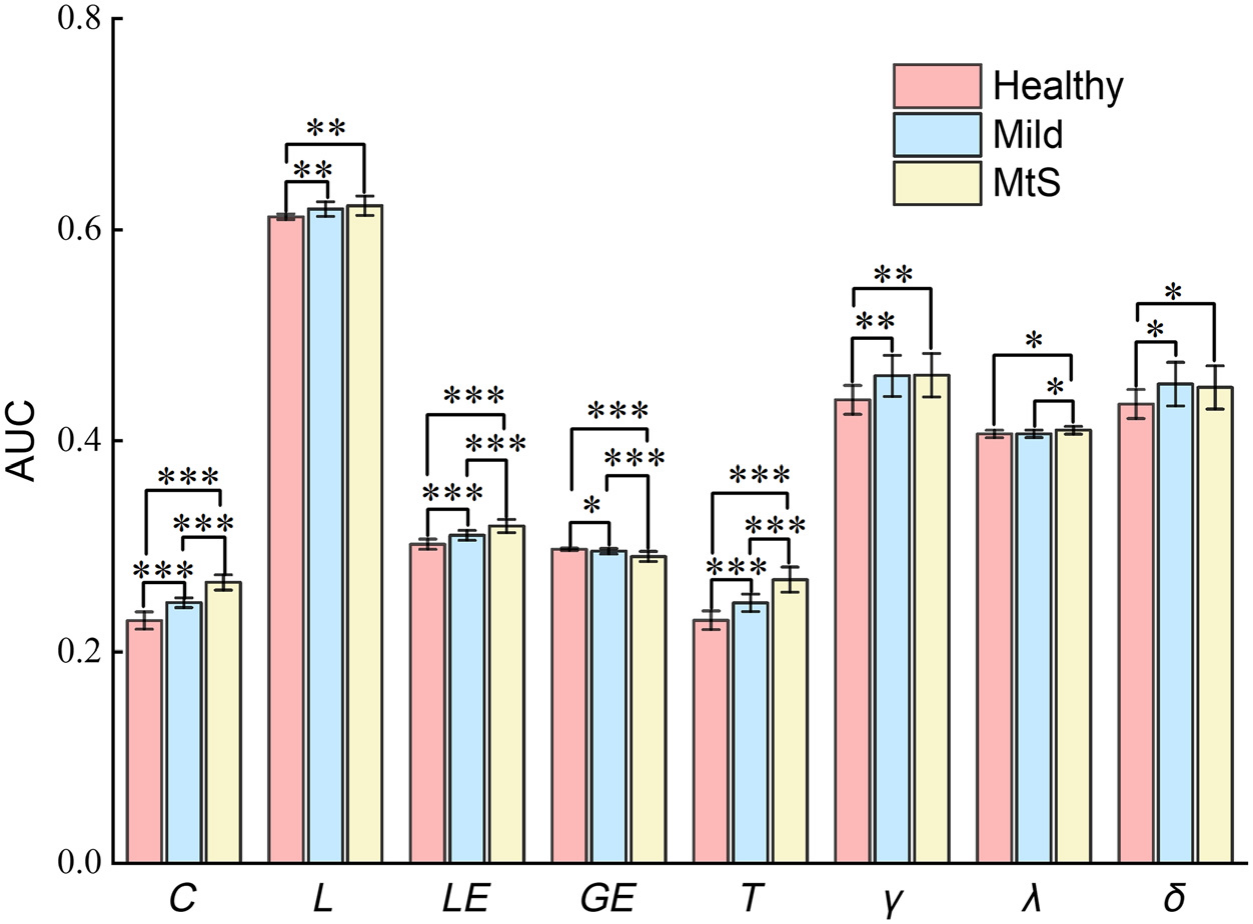
AUC indicators of the small-world properties for the three groups, * indicates p < 0.05, ** indicates p < 0.01, and *** indicates p < 0.001 (LSD correction).

ANOVA with age and gender as covariables revealed that the group effect was significant for the AUCs of $C(p={1e}^{-13})$, $L(p={7e}^{-3})$, $LE(p={2e}^{-7})$, $\mathrm{GE}(p={3e}^{-4})$, $T(p={2e}^{-9})$, and γ(p=0.019). Then, we performed posthoc T-tests to analyze AUC differences between groups, and the results are shown in Fig. 4.

### 3.3 Relation Between Network Properties and Patients’ Motor Function

**Fig. 5:**
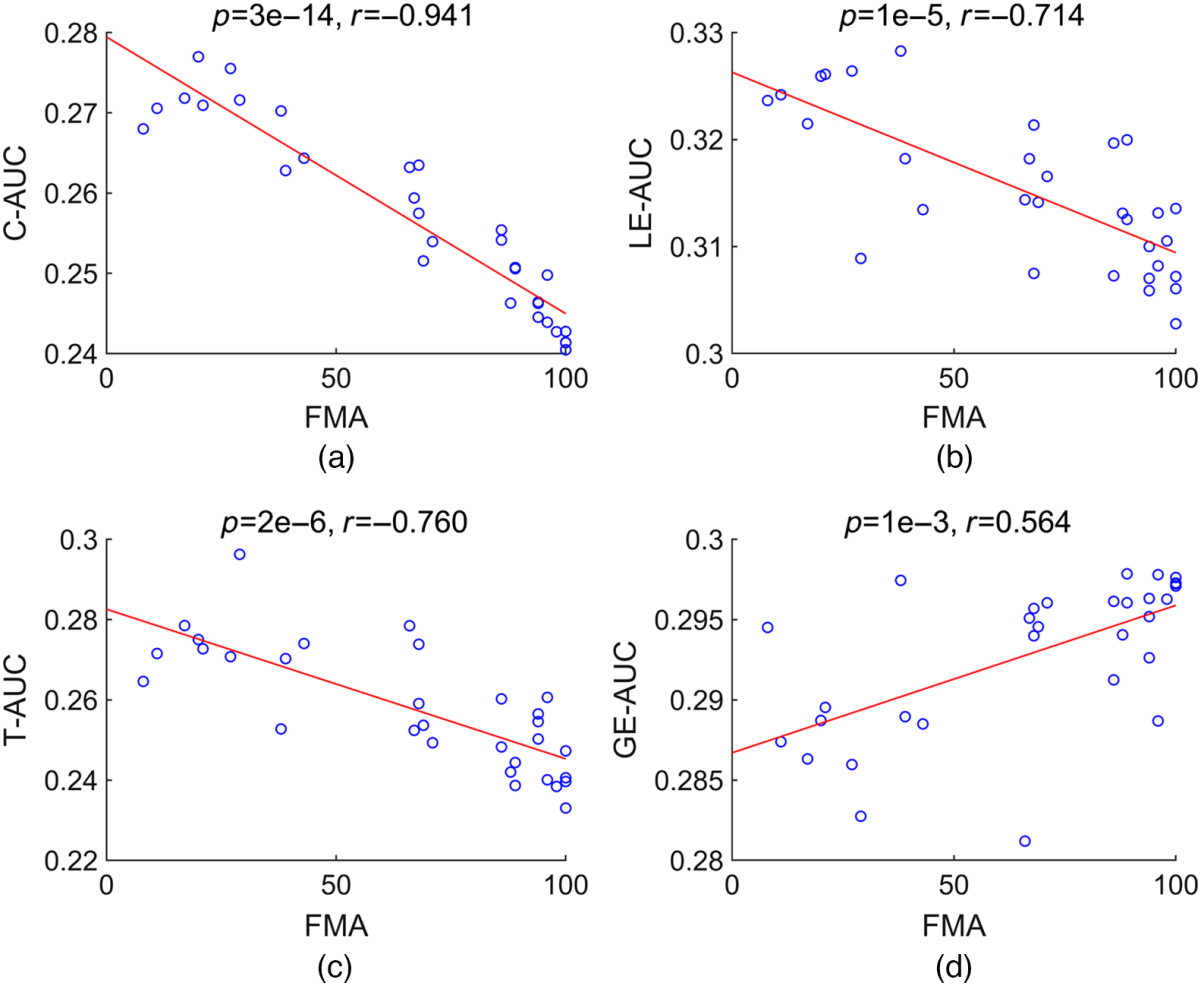
Scatterplots of significant correlations between FMA and C-AUC (a), LE-AUC (b), T-AUC (c), and GE-AUC (d).

With age and gender as control variables, we conducted Partial correlation analyses between patients’ FMA and the AUCs of eight network properties. The results displayed that AUCs of C, LE, and T had a significant negative correlation with FMA, whereas the AUC of GE had a significant positive correlation with FMA (Fig. 5).

### 3.4 SVM Classification

**Fig. 6:**
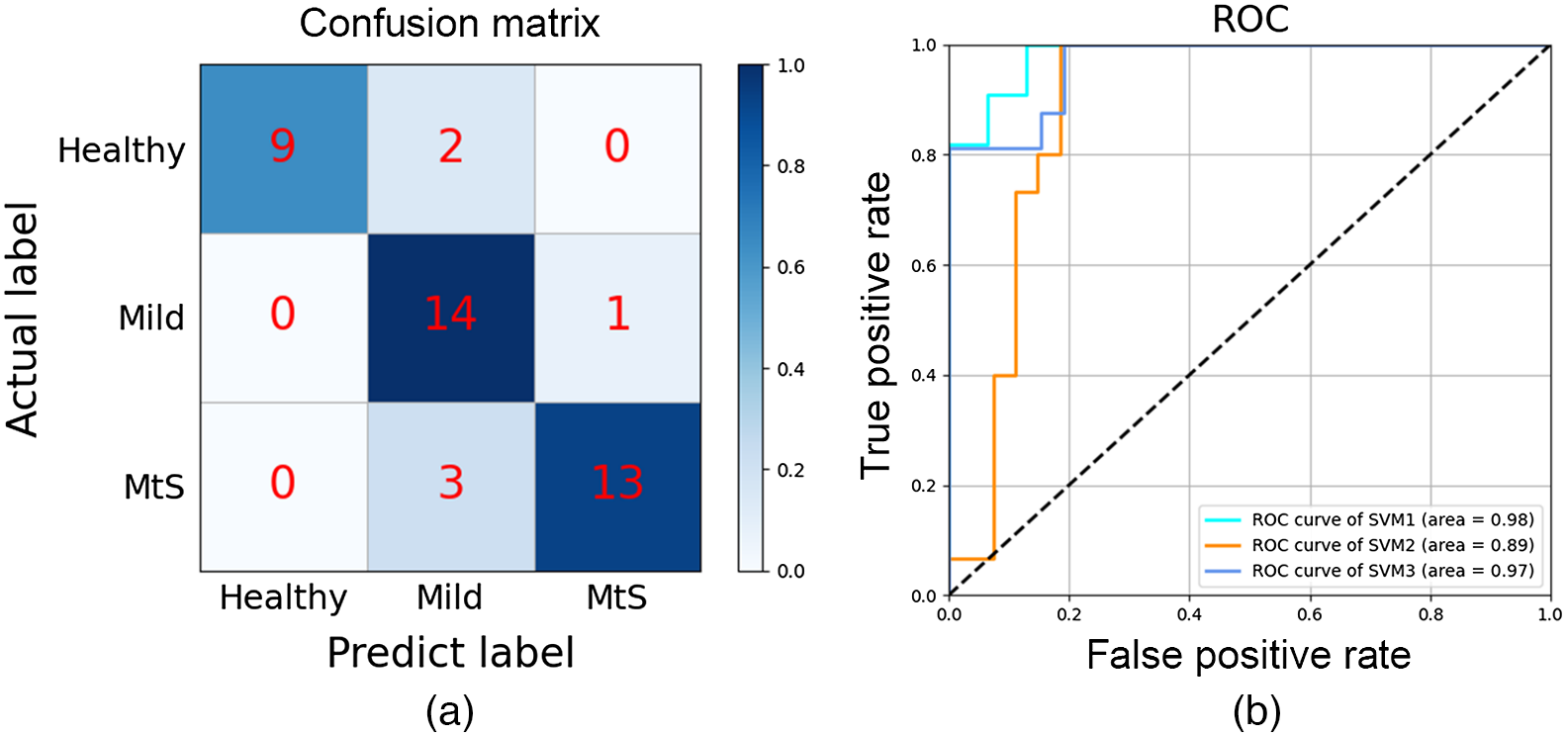
(a) The confusion matrix of the classification result and (b) the ROC curves of the three SVM models.

We used AUCs of all topological properties as alternative characteristics, and the results of feature selection showed that C (weight = 0.232) presented the highest distinguishing ability, followed by LE (weight = 0.222), T (weight = 0.162), L (weight = 0.120), GE (weight = 0.110), γ (weight = 0.103), δ (weight = 0.038), and λ (weight = 0.013) in sequence. The established SVM model achieved the highest accuracy of 85.7% for classifying the three groups of subjects using the first six network properties’ AUCs as features. The receiver operator characteristic (ROC) curves of the SVM models are shown in Fig. 6(b). The AUCs of ROC curves of SVM1 (Healthy to others); SVM2 (Mild to others) and SVM3 (MtS to others) were 98%, 89%, and 97%, respectively; the sensitivities of SVM1, SVM2, and SVM3 were 81.8%, 93.3%, and 81.3%, respectively; and the specificities of SVM1, SVM2, and SVM3 were 100%, 88%, and 95.8%, respectively. The small-world properties demonstrated high sensitivity and specificity for Healthy, Mild, and MtS subjects.

## 4 Discussion

In this study, we used the graph theory approach to study the RS motor network reorganization in stroke patients and attempted to establish a machine learning-based assessment model to predict patients’ motor dysfunction at the individual level. The motor network’s small-world properties were significantly different between healthy subjects and stroke patients as follows: (1) C, LE, and T: MtS > Mild > Healthy; (2) GE: MtS < Mild < Healthy. C, LE, T, and GE all correlated with patients’ FMA scores. Using the small-world properties, we constructed SVM models that could differentiate among the three groups of subjects with a classification accuracy of 85.7%.

After a stroke, the neurological disconnect may result in function reorganization of the whole brain network.^45^^,^^49^ Even though most stroke patients did not have a lesion in the motor cortex, their motor network differed from healthy subjects. Stroke patients’ C and T increased (MtS > Mild > Healthy), indicating an increase in the amount of local short connections and the emergence of high local clustering in patients’ motor network, which resulted in an increase of LE (MtS > Mild > Healthy). When patients’ motor function got worse, this phenomenon became more serious. Stroke patients had a higher L (MtS, Mild > Healthy), which indicated that the motor network’s capacity for integrating and transmitting information became worse for patients,^50^ which resulted in a decrease of GE (MtS < Mild < healthy). Yin et al. found an increase of LE and a decrease of GE in the motor-related cortical network for stroke patients with motor pathway damage compared with healthy controls.^51^ Our results are consistent with their research. In addition, we found that this network property change was present in both the MtS and Mild groups. The patients’ higher LE in the motor network might be a defense mechanism in response to motor injury.^52^ The stroke patients also had a higher γ and δ (γ and δ: MtS, Mild > Healthy > 1), which suggested that the structure of the motor network shifted toward a regular network. On the other hand, there was no significant difference in the normalized path length (λ≈1) between the three groups, indicating that the reorganization of the motor network may primarily involve separation rather than integration.^53^

Machine learning has been widely used in medicine due to its ability to identify discriminant variables that can be used for prediction^54^ and easily incorporate new data to improve prediction performance.^55^ Machine learning has improved the assessment and prediction for both diagnosis and treatment purposes in stroke research.^56^^,^^57^ The goal of this study was to develop a machine learning-based assessment approach to provide an objective evaluation of stroke patients’ motor function. According to the selected topological properties, the established SVM models were able to categorize the three groups of subjects with different motor function capacities with high accuracy. Any translational implementation should take into consideration the fact that the cost of misclassifying an ill person as healthy is greater than the cost of misclassifying a healthy person as ill.^25^ Our OVR-based classifiers demonstrated high sensitivity and specificity, with 100% specificity for SVM1 classifying healthy controls and stroke patients [Fig. 6(a)], which indicated that no stroke patients had been misclassified as healthy. Additionally, the motor network properties (C, LE, T, and GE) linearly correlated with patients’ FMA. It has been proved that stroke patients’ motor performance was more accurately classified by M1 RS connectivity than by lesion location.^28^ These all point to the effectiveness of using the RS motor network as a diagnostic marker to gauge the poststroke dyskinesia degree.^58^

Because the RS is task-free, stroke patients with severe dyskinesia can also be included to carry out automated assessments,^59^^,^^60^ significantly expanding the application scope of our method. NIRS is portable, low-cost, and invasive.^61^ Our assessment method can be used repeatedly throughout a patient’s rehabilitation process. NIRS devices that are wireless and wearable have been developed.^62^ They facilitate the rehabilitation follow-ups within home and community settings and allow doctors and physicians to track a patient’s functional status and provide timely, accurate and targeted treatment plans.^63^^,^^64^ The development of NIRS and the proposed new indicators will undoubtedly promote the use of NIRS in clinical applications. It is anticipated that our method could be adapted to other diseases with dyskinesia, such as Parkinson.^65^^,^^66^

There are also some limitations in this study. First, the sample size was small. However, the post-hoc tests showed that the differences in small-world properties between the three groups were reliable. Second, the lesion location and size were not consistent in patients. The time from the stroke onset to NIRS measurement varied widely among patients. These factors may increase the RSFC measurement variability. In future studies, we will recruit more patients and further validate our method by dividing patients into specific subgroups according to the lesion site and FMA scores. Third, the age differed between groups. But the age factor was treated as a control variable in the ANOVA and correlation analyses, and the regression analysis results of age and each network property’s AUC showed no significant correlation. We will match age and gender between different groups, because there might be a more complex relationship (rather than a simple linear correlation) between demographic factors and network properties. So, it would be better to match age and gender between different groups. Finally, Pearson correlation was used to define the strength of functional connectivity. Previous studies have shown that the small-world properties obtained from Pearson’s correlation exhibit high reliability when the length of data exceeds 2.5 min.^67^ The data length selected for analyzing the small-world properties in this study can ensure the stability of network properties. Other methods (such as coherence analysis and mutual information) can be used in the future to provide more information on brain connectivity, which would reduce the error rate and improve inferences about underlying neural mechanism.^68^

## 5 Conclusions

This study proved the motor network reorganization and its correlation with motor function in stroke patients: more serious dyskinesia is associated with larger C, LE, and T and smaller GE. The combination of NIRS, RSFC, and SVM constitutes a sensitive technique for assessing stroke patients’ dyskinesia degree at the individual level. All data were acquired during a routine imaging session, highlighting the clinical viability of our method in patients with acute and severe motor disorders. Our study has great significance for the immediate evaluation of motor function during rehabilitation, which could be done repeatedly, and the guidance of personalized rehabilitation programs; it can also reduce the workload of clinicians.

## Code, Data, and Materials Availability

Data are available on request due to privacy/ethical restrictions.
