# Supplementary material for: Predicting poststroke dyskinesia with resting-state functional connectivity in the motor network
Source: Neurophotonics. 2023 Apr 4;10(2):025001. doi: 10.1117/1.NPh.10.2.025001 (PMC10072005; doi:10.1117/1.NPh.10.2.025001)
Supplement: Supplementary file 1 [file NPh_010_025001_SD001.pdf]

Appendix A: Supplemental Material

A.1 the results and figures of Hb

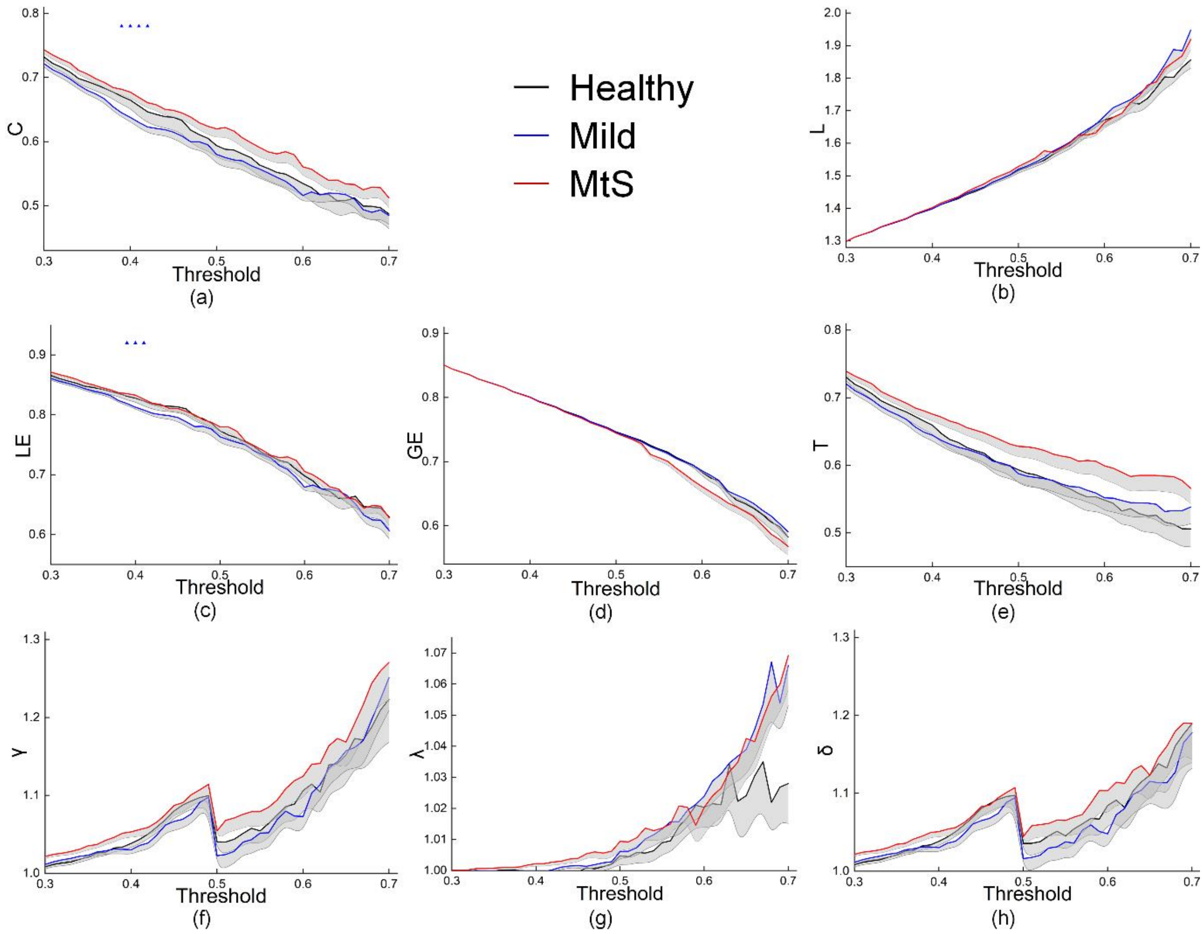

Fig. S1. Hb: The mean C (a), L (b), LE (c), GE (d), T (e), γ (f), λ (g) and δ (h) of Hb data for the three groups under each threshold. The shaded part indicates the standard error (SEM). The black, blue and red triangles represent significant differences between Healthy and Mild groups, Mild and MtS groups, and Healthy and MtS groups ($p < 0.05$, FDR correction) under this threshold, respectively.

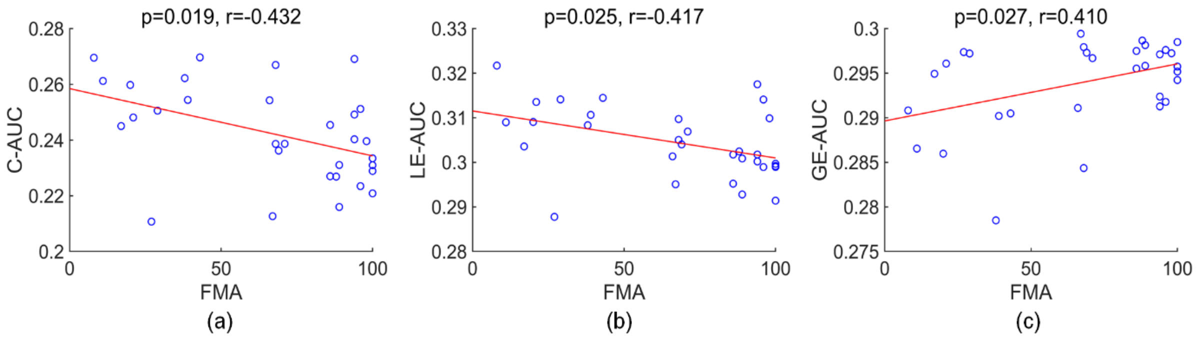

Fig. S2. Hb: Scatterplots of significant correlations between FMA and the AUCs of C (a), LE (b) and GE (c).

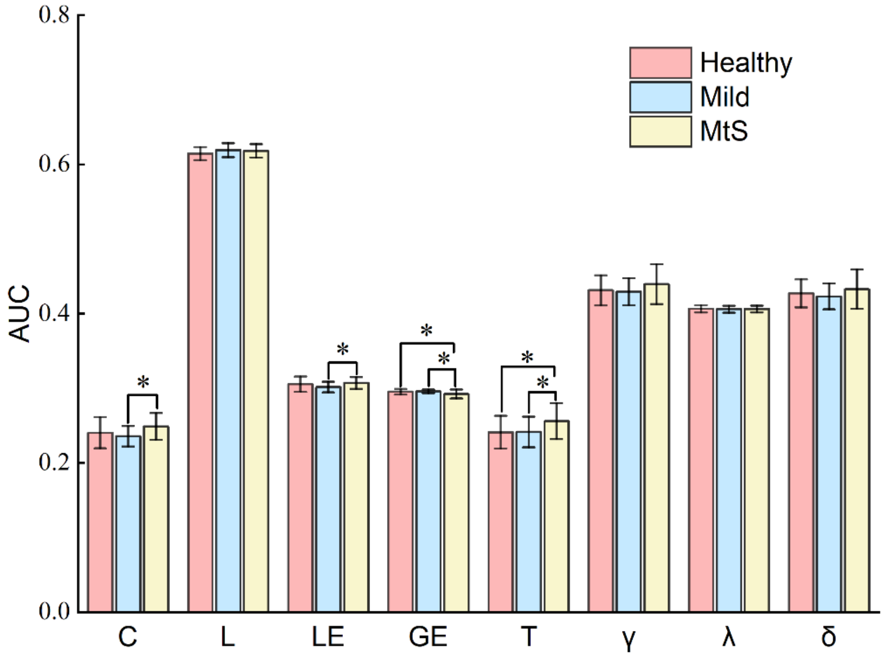

Fig. S3. Hb: The AUC indicators of the eight small-world properties for the three groups, * indicates $p < 0.05$ (LSD correction).

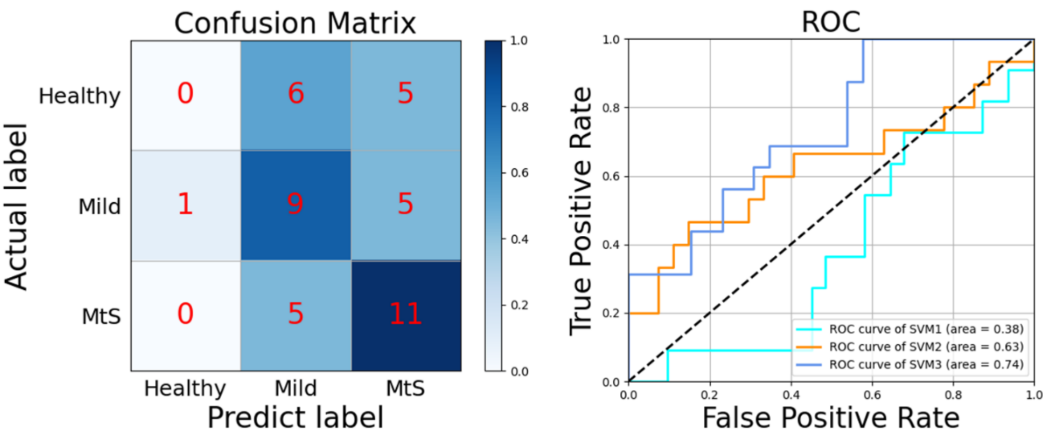

(a) (b)

Fig. S4. Hb: (a) The confusion matrix of the classification result; (b) The ROC curves of the three SVM models.
